# The Relationship between Emotional Stability, Psychological Well-Being and Life Satisfaction of Romanian Medical Doctors during COVID-19 Period: A Cross-Sectional Study

**DOI:** 10.3390/ijerph19052937

**Published:** 2022-03-02

**Authors:** Lorena Mihaela Muntean, Aurel Nireștean, Cosmin Octavian Popa, Elena Gabriela Strete, Dana Valentina Ghiga, Andreea Sima-Comaniciu, Emese Lukacs

**Affiliations:** 1 Department of Psychiatry, “George Emil Palade” University of Medicine, Pharmacy, Sciences and Technology of Târgu Mureș, 540136 Târgu Mureș, Romania; lorena-mihaela.muntean@umfst.ro (L.M.M.); elena.buicu@umfst.ro (E.G.S.); emese.lukacs@umfst.ro (E.L.); 2 Department of Ethics and Social Sciences, “George Emil Palade” University of Medicine, Pharmacy, Sciences and Technology of Târgu Mureș, 540136 Târgu Mureș, Romania; 3 Department of Medical Scientific Research Methodology, “George Emil Palade” University of Medicine, Pharmacy, Sciences and Technology of Târgu Mureș, 540136 Târgu Mureș, Romania; dana.ghiga@umfst.ro; 4 Doctoral School of “George Emil Palade” University of Medicine, Pharmacy, Sciences and Technology of Târgu Mureș, 540136 Târgu Mureș, Romania; andreea.comaniciu@umfst.ro

**Keywords:** psychological well-being, emotional stability, distress, medical doctors, life satisfaction

## Abstract

Due to the COVID-19 pandemic, as well as the fast progression of modern society, occupational stress has recently reached alarming levels with consequences for doctors’ psychological well-being. The aim of this study was to analyze the relationship among emotional stability, psychological well-being, and life satisfaction of medical doctors. We conducted a cross-sectional study on 280 medical doctors from Romania between February 2021 and September 2021, in the period between the third and fourth pandemic waves, who were evaluated by the DECAS, ASSET, and Satisfaction with Life scales. Our results showed that emotional stability is negatively correlated with psychological well-being (*r* = −0.526, *p* < 0.000) and positively correlated with life satisfaction (*r* = 0.319, *p* < 0.0001). Between psychological well-being and life satisfaction, we found a negative correlation (*r* = −0.046, *p* < 0.001). This study shows that there is a correlation among emotional stability, psychological well-being, and life satisfaction, which is why it can be considered that Romanian doctors have generated coping mechanisms during the COVID-19 pandemic.

## 1. Introduction

Stress is a widely studied concept and refers to how each of us responds positively or negatively to an internal or external stimulus/condition that often exceeds perceived coping abilities [1]. These stressors can be divided into three categories: circumstantial, occupational, and personal [2]. Occupational stress in the medical world is a global phenomenon faced by modern society, negatively affecting both the physical and the mental health of the physician, followed by consequences for the quality of the medical act to the detriment of the patient [3]. Physicians must constantly face high standards at the workplace, and they frequently face problems such as lack of time due to the increased number of patients, inability to cope with situations due to a lack of skills needed in the specialty they practice, as well as a lack of support from their colleagues [4], and increased number of hours spent in the hospital, especially night shifts with sleep deprivation [5]. Therefore, there are other significant aspects that result in a decrease in the free time intended for the physician’s recovery, as well as increased family problems and financial issues. Given the current situation, it has been demonstrated that the pandemic of coronavirus disease 2019 (COVID-19) has raised distress for medical doctors; in addition to all the factors mentioned above, it is important to underline the risk for this professional category to develop different mental and physical disorders, in this pandemic context [6,7]. Furthermore, during the pandemic period, medical doctors presented a higher risk of developing depression and/or anxiety disorders compared to other professions [8]. Moreover, compared with other medical specialties, this risk is higher especially for the doctors who work on the front line treating COVID-19 patients [9]. Other factors that contribute to elevated stress levels in doctors in the pandemic context include a lack of protective equipment [10], overloading during busy periods with many patients [11], the fear of becoming infected, and concern regarding the possibility of infecting family [12], as well as the possibility of friends and relatives avoiding being in the presence of medical staff [13]. Stress levels are used as an indicator in mental health, and more and more studies have shown that employment and labor conditions in the medical field affect the psychological well-being (PWB) of healthcare employees [14,15]. All of these factors increase stress, eventually leading to the development of burnout syndrome [16]. Burnout syndrome has increased in recent years among physicians [17] and is defined as the response to prolonged exposure to high levels of stress at work. It is manifested by emotional exhaustion, episodes of depersonalization, and decreased performance at work [18]. The factors that contribute to the increase in occupational stress consist of personality traits, stressors related to patient wellbeing, level of experience, and attitude at work [19,20]. Occupational stress and involvement at work are negatively correlated, whereby doctors who have a high level of stress tend not to get involved as much as others [21]. Lack of control at work increases the level of occupational stress. Workplace performance is influenced by the doctor’s experience and by job stability [22].

Both stress factors and daily challenges influence the doctor’s PWB. The most pressing aspects are related to the doctor’s responsibility for the patient’s wellbeing, the lack of control related to the patient, the high standards that patients and relatives have, and their dissatisfaction with the medical act [23].

PWB is a construct consisting of several dimensions according to the study conducted by Ryff and Keyes (1995): self-acceptance refers to the acceptance of one’s own person with advantageous but also disadvantageous personal traits, including acceptance of one’s own past; personal growth refers to continuous development by experiencing new things; purpose in life refers to the belief that everyone’s life has a purpose which is significant; positive relationships with others; environmental mastery through which everyone has the ability to manage and route things to the desired direction; autonomy, i.e., the determination that each of us has in achieving the proposed goals [24,25]. PWB refers to both continuous personal development and the concept of living well and being well with oneself [26].

Subjective wellbeing refers to the satisfaction and happiness that each of us feels, encompassing a cognitive component and an affective one [26]. The cognitive component is the evaluation of life satisfaction (LS), while the affective component is represented by positive and negative affect [27]. They integrate the levels of individual satisfaction into life roles [28]. Furthermore, PWB is closely related to both job satisfaction and LS. A study conducted in Denmark on a large number of general practitioners showed that one in five doctors faces a high level of stress and low PWB [29]. A recent study on family physicians from China showed that the level of involvement in the workplace is positively correlated with high performance, while PWB is positively correlated with high performance. Fulfilled, happy, and motivated family physicians who identify themselves with the environment where they work and keep proper relationships with their colleagues have increased PWB [30]. Recent research in China showed that the level of PWB is positively correlated with the title of physician and negatively correlated with age and education [31]. PWB is most often associated with personality traits [32].

From the dimensional perspective of personality assessment, the emotional stability (ES) dimension, the fifth dimension of the Big Five Model (FFM = Five Factor Model) is most often associated with subjective wellbeing [33]. People with high ES are characterized as relaxed, emotionally stable, resilient, optimistic, and rational in thinking. On the other hand, people with low ES are characterized as anxious, scared, and easily irritable, with a low tolerance for frustration and an amplified response under stress [34]. 

Rus et al. highlighted the fact that there is a positive correlation between subjects who obtained low scores in the ES dimension and high levels of stress among medical employees [14]. Low ES was associated with a risk factor for the inability to manage a work–life balance by physicians from all specialties [35]. Furthermore, low ES was positively associated with the emotional exhaustion dimension specific to the burnout syndrome in medical staff from private hospitals from India [36]. Moreover, it is important to mention that, during the COVID-19 pandemic, low levels of ES and extraversion were the main personality dimensions, from the Big Five Model, related to high distress and fear in both the general population and healthcare workers [6,37].

LS is defined as the evaluation of one’s own life, as well as the way one feels depending on the objectives or the things that can be obtained in the future [38]. It can also be defined as one’s judgment according to one’s own balance, expectations, or standards [39,40]. Regarding the factors that influence LS, it has been shown that work–life imbalance, multiple tasks at work, and a lack of support from colleagues negatively affect LS [41]. Factors that positively influence LS are represented by adequate working hours, proper physical health, the existence of the necessary resources for patient care, and working for more than 4 years at the same job [42]. It has also been shown that personality traits have an important role in LS [43]. It has been demonstrated that ES is positively correlated with LS [44].

ES has been described as a factor of resilience to psychological distress in medical workers, during the COVID-19 pandemic [8,45,46]. In addition to ES, another adaptive coping mechanism with stress is represented by PWB [47]. What is important to find out in this context is whether these two psychological dimensions constitute just a resilient factor, as already demonstrated by previous studies, or whether these psychological dimensions are correlated with satisfaction in life, in Romanian medical doctors during the COVID-19 pandemic.

In light of the evidence discussed above, the aim of this study was to establish whether there is a correlation among ES, PWB, and LS in Romanian medical doctors, in the period between the third and fourth pandemic waves of COVID-19. In this context, we hypothesize that the association among ES, PWB, and LS present in Romanian doctors generated different adaptative coping mechanisms, in the period between the third and fourth pandemic waves of COVID-19.

## 2. Materials and Methods

This was a cross-sectional study conducted between February 2021 and September 2021 at the “George Emil Palade” University of Medicine, Pharmacy, Science and Technology of Târgu Mureș on physicians who graduated in medical studies and carried out their medical activity in Romania. Due to the fact that the study took place in the period between the third and fourth pandemic waves of COVID-19, it is important to mention that medical doctors included in this study were selected from the second line of COVID-19 treatment. This research was approved by the Ethics Commission of the “George Emil Palade” University of Medicine, Pharmacy, Science, and Technology of Târgu Mureș by decisions no. 1250/28.01.2021 and 1374/20.05.2021. All participants signed the informed consent form before enrolling in the study.

### 2.1. Participants and Procedure

Out of an initial 311 subjects, 280 participants who met the eligibility criteria were included in this study. The sample of the present study was constituted using a simple sample randomization. Therefore, our study group was considered a representative one according to the total number of Romanian doctors working in Romania. According to the official date of Ministry of Health, about 63,000 medical doctors are active in Romania [48]. In rapport with the simple sample calculation method, this study would need to include 245 medical doctors for a representative sample population (representing 80% of the number of doctors from the second line of treating COVID-19, 95% CI). Therefore, in our sample we included 280 medical doctors, and this sample can be considered representative of Romanian medical doctors from the second line of treating COVID-19. Furthermore, 30 subjects were excluded due to the fact that they did not pass the internal validations scales of the DECAS Personality Inventory, while one subject did not carry out activities in Romania. The subjects completed the following scales: DECAS Personality Inventory, ASSET (A Shortened Stress Evaluation Tool), and the Satisfaction with Life Scale. In addition to the applied scales, the following parameters were included in the analysis: age, sex, level of experience. Inclusion criteria were as follows: (1) doctors who carried out medical activity in Romania; (2) doctors who graduated from the Faculty of General Medicine in Romania. Exclusion criteria were as follows: (1) doctors who did not carry out medical activity in Romania; (2) doctors who did not pass the internal validation scales of the DECAS Personality Inventory; (3) doctors who worked on the front line in the fight against the pandemic.

Prior to enrolling in the study, all participants signed the informed consent form. The questionnaires were disseminated online through social media to medical groups from Romania.

### 2.2. Measures

To evaluate the subjects, we administered three scales validated on the Romanian population: the DECAS Personality Inventory (DECAS), A Shortened Stress Evaluation Tool (ASSET), and the Satisfaction with Life Scale (SWLS).

The DECAS Personality Inventory (DECAS) is a personality assessment scale based on the FFM, developed by Sava et al. [49]. It is a scale consisting of 97 statements for the assessment of each personality dimension: openness, extraversion, conscientiousness, agreeableness, and emotional stability. The openness dimension, the least studied dimension in the literature, is assessed through 18 items and a reserve one that targets the following facets: fantasy, aesthetics, feelings, actions, intellectual curiosity, and values. The extraversion dimension is the most obvious dimension and, along with the emotional stability dimension, is found in the descriptions of all reference models of personality assessment, consisting of the following facets: warmth, sociability, assertiveness, activism, sensation seeking, and positive emotions. The conscientiousness dimension includes the following six facets: competence, order, sense of duty, desire to achieve, presumption, and deliberation. The agreeableness dimension has the greatest impact on interpersonal relationships, consisting of the following facets: trust, direct behavior, altruism, goodwill, modesty, and gentleness. The emotional stability dimension includes the following facets: anxiety, anger, depression, self-awareness, impossibility, and vulnerability. In addition, the DECAS personality inventory includes three validation scales built with the purpose of evaluating the sincerity of the answers of the subjects: social desirability (SD), random answers (RD), and approval (AP). The SD validation scale is a factor that measures the tendency of the subjects to put themselves in a favorable light through the answers offered in the questionnaire items. A score of more than 65 (*T*-scores) obtained by the subject on this scale invalidates the results. The RD validation scale represent a factor which evaluates the subject’s tendency to give random answers, whereby a score higher than 70 points (*T*-scores) on this scale leads to the invalidation of the personality inventory protocol. The AP validation scale is a sensitive factor to the subject’s tendency to respond more with “true” or “false”, and a score of more than 65 points (*T*-scores) or a score of less than 35 points (*T*-scores) invalidates the protocol. Regarding the inventory psychometric properties, internal consistency was calculated following the assessment of a batch of 1524 people with alpha Cronbach coefficient values assessed on the Romanian population ranging from 0.70 for the conscientiousness dimension to 0.75 for the emotional stability dimension. The Cronbach’s alpha internal consistency coefficient was 0.69 for SD and 0.71 for AP [49].

A Shortened Stress Evaluation Tool (ASSET) was developed by Cooper and Cartwright, which can be easily used to identify potential stress exposure for employees in various fields [50,51]. The tool measures the following variables as stressors: professional relationships, work–life balance, overload, workplace safety, environmental control, access to resources and communication, and payments and benefits. The second section defines the perception of the involvement level both as an employee of the respective institution and as the involvement level of the institution toward the employee. The third section investigates stress effects on physical health and mental wellbeing, and the fourth section focuses on job aspects related to job satisfaction or physical job conditions [50,51]. The instrument has a total of 63 items scored on a six-point Likert scale and 37 items for biographic data (current job, family, education, lifestyle, and interests). The 63 items are distributed in several subscales aimed at professional relations, work–life balance, overload, workplace safety, control, resources and communication, payments and benefits, work aspects, the perceived commitment of the organization toward its employees, the commitment of members toward their organization, PWB, and physical health. For some dimensions of the scale, for example, PWB, the interpretation of the results is made in the opposite way. The score of this subscale is interpreted as follows: <3—very good level of PWB, <4—good level of PWB, 4–7—medium level of PWB, >7 low level of PWB, >8 very low level of PWB. Therefore, lower scores obtained by the subject for PWB can be interpreted as reduced distress levels for the subject and a good PWB. In terms of internal consistency, the alpha Cronbach coefficient measured on the Romanian population showed an average of 0.73 across all scales, with only two subscales below 0.60 [50]. 

The Satisfaction with Life Scale (SWLS) was developed by Diener et al. and is a measuring instrument designed to assess subjective wellbeing, from the perspective of its cognitive component. It consists of five questions scored on a seven-point Likert scale. The score of the scale is interpreted as follows: 31–35—extremely satisfied, 26–30—satisfied, 21–25—slightly satisfied, 20—neutral, 15–19—slightly dissatisfied, 10–14—dissatisfied, 5–9—extremely dissatisfied. The alpha Cronbach coefficient assessed on the Romanian population was 0.82, proving its good internal consistency [52].

### 2.3. Statistical Analysis

Statistical analysis was performed using the GraphPad Prism 7 licensed software. The significance level for the *p*-value was set to 0.05, with a confidence interval CI = 95%. Statistical analysis included elements of descriptive statistics (mean, median, standard deviation) and elements of inferential statistics. To determine whether there was a statistically significant difference between the median values of ES, PWB, and LS in resident doctors and senior doctors, we applied the Mann–Whitney test for unpaired data. To determine the distribution of data series, we applied the Shapiro–Wilk test. The Spearman test, a nonparametric test, was applied to measure the strength and direction of the association among the studied variables (ES, PWB, and LS). 

## 3. Results

Out of an initial number of 311 subjects, 280 participants who met the eligibility criteria were included in this study, of whom 233 (83.21%) were female and 47 (16.79%) were male. The mean age of the group was 28.81 ± 4.79 years. Regarding experience, 33 (11.78%) were senior doctors and 247 (88.21%) were junior doctors. The distribution in the sample included the follows categories: medical specialties 191 (68.22%), surgical specialties 39 (13.93%), and paraclinical specialties 50 (17.85%). Demographic characteristics are summarized in Table 1.

Descriptive statistics (Table 2) revealed that the doctors had a medium level of PWB (6.08 ± 2.06), and they were satisfied with life (27.02 ± 5.49). Moreover, the level of the ES (46.28 ± 8.78) dimension was medium.

Regarding the implications of ES, our results showed a significant negative correlation between ES and PWB (*r* = −0.526, *p* < 0.0001). Furthermore, there was a significant positive correlation between ES and LS (*r* = 0.319, *p* < 0.0001). Between LS and PWB, we found a significant negative correlation (*r* = −0.046, *p* < 0.001). The correlations among the three variables are found in Table 3. 

## 4. Discussion

The present study investigated the correlations among ES, one of the dimensions of the Big Five Model, PWB, and LS in medical doctors in the period between the third and fourth pandemic waves of COVID-19.

Given that stress levels were high during the pandemic, our study shows that physicians had a moderate level of PWB, and they were satisfied with life. These results are consistent with previous studies [53,54]. These aspects may be due to the fact that the study was conducted in the period between the third and fourth pandemic waves, at which point the doctors had become familiar with the pandemic, and things had become clearer [55]. Longitudinal studies have shown that resilience has increased and the general population has found a surprising ability to adapt [56,57]. Coping mechanisms such as active attitudes, along with making plans, acceptance, and reinterpretation of reality are positively associated with LS [58]. Other factors that have helped to reduce stress are protective measures, psychological counselors [59], team support, stress monitoring, taking breaks regularly [60], knowledge of the disease [61], and things becoming easier [62].

ES is an independent predictor of LS [41,63]. In our study, the ES dimension was positively correlated with LS. In the literature, there are other similar positive correlations between ES and LS, regardless of the scale or questionnaire applied for personality evaluation [64,65]. This finding is identical to that of Tyssen et al., according to whom doctors with low ES responded excessively to the stressful conditions imposed by their profession with implications for daily activities [66]. During COVID-19, levels of stress were higher, associated with avoidance of the use of coping mechanisms [67], but people with high ES could overcome these issues by following doctors’ recommendations [68]. Moreover, low levels of anxiety facilitate adaptability in all existential roles [69].

We found that, between LS and PWB, there was a negative correlation (lower levels on the scale indicating reduced distress levels for the subject and a good PWB).

This is confirmed by several studies that showed a relationship between LS and stress or other constructs such as PWB [70,71]. A possibility to increase the level of PWB is by using approach-oriented coping strategies, because they are connected with a higher level of PWB [72]. At the same time, LS, understood as the achievement of goals, leads to beneficial cognition and is negatively correlated with avoidance coping strategies [73].

Our results indicated that the ES dimension was negatively correlated with the PWB of the doctor (lower levels on the scale indicating reduced distress levels for the subject and a good PWB). These observations are also supported by the results of the study by Soh et al., which stated that emotional stability is an important predictor of PWB [74,75]. A relaxed doctor, who controls the situation, with good emotional control and stress resistance, will have a better PWB, which is also reflected by involvement in the professional role [76].

Psychological resilience implies maintaining a consistent level of happiness and PWB in the face of stressors. This means developing strategies in work over the years in order to conserve a good mental health. This can be translated into practices and behaviors that the physicians consider being good to protect their PWB [77]. A low level of PWB manifested by depression and anxiety has direct effects on choosing avoidance coping strategies instead of applying problem-focused strategies [78].

The use of an adaptative coping mechanism/resilience during COVID-19 is influenced by both EA and PWB [79]. A comparative study of resident and senior doctors showed that both categories used coping mechanisms during the pandemic. These mechanisms varied with age, whereby resident doctors were more technology-oriented and practiced more mindfulness than senior doctors [80]. Resilience is associated with maturity, responsibility, optimism, perseverance, and cooperation [81]. Physicians usually present increased resilience through their education, which is necessary to cope with the daily challenges of their chosen profession, especially in the COVID-19 pandemic [82]. Stress can be overwhelming regardless of the level of experience throughout the medical profession [83]. It has been shown that subjective wellbeing and satisfaction with life have an important impact on improving physicians’ resilience to stress [84]. In this regard, the level of resilience could be increased by an improvement of ES, PWB, and LS [85]. From this point of view, resilience may be the crucial aspect to focus on when elaborating programs to support mental health [86]. Although ES is a personality trait, personality is a construct that is relatively stable over time with small changes over short periods [87]. It has recently been shown that there is a significant difference in the stability of personality traits between adolescence and adulthood [88]. Accordingly, there is the possibility that practicing therapy to learn techniques can lead to an increase in emotional stability over time [89]. Hypnotherapy combined with behavioral cognitive therapy (CBT) has been shown to significantly improve emotional stability [90]. Another approach is to restore the balance between mind and body by practicing mindfulness [91,92]. Healthcare workers experience increased levels of daily stress. During the pandemic, these concerns have been at a higher level due to overload at work, the fear of infecting both themselves and their relatives, and high levels of uncertainty about the future of the pandemic [93]. Doctors have developed coping mechanisms through social distancing, wearing a mask, collaborating with colleagues to manage patients, and recurrent training and pandemic information received from the institution where they practice [94]. Acceptance and engagement therapy (ACT) is an acceptance-based behavioral intervention that promises to reduce the psychological impact of the pandemic. The ACT increases both behavioral awareness and openness to experience. Through ACT, the doctor takes on the role of observer of their own thoughts [95]. ACT is used to improve the functioning of the workplace, to reduce the stress caused by daily activities, and to improve relationships with others [96].

## 5. Limitations

Our study had some limitations that deserve attention in the future. The first limitation is that we used self-administered questionnaires that could have contributed to inaccurate results due to the fact that only the DECAS Personality Inventory has an internal validation scale that can detect distorted responses. It is recommended that future studies be conducted in this direction to figure out which of the variables (ES, PWB, and LS) are interrelated. Furthermore, in the future, the level of resilience/coping mechanisms can be assessed in terms of a correlation with ES, PWB, and LS.

## 6. Conclusions

ES and PWB were found to be correlated with LS; thus, it can be considered that Romanian doctors generated coping mechanisms during the COVID-19 pandemic. In addition, the level of emotional stability and psychological well-being of the doctors was moderate, and they perceived an increased level of life satisfaction in the period between the third and fourth pandemic waves, confirming that coping mechanisms were generated to deal with the pandemic. Future research may investigate these coping mechanisms.

## Figures and Tables

**Table 1:** Demographic characteristics of participants.

Sample Characteristics	*N* = 280
Gender, *n* (%)	
Female	233 (83.21)
Male	47 (16.79)
Age range, M (SD)	25–58
	28.81 (4.79)
Experience, *n* (%)	
Senior	33 (11.78)
Junior	247 (88.21%)
Specialty, *n* (%)	
Medical	191 (68.22)
Surgical	39 (13.93)
Paraclinical	50 (17.85)

Legend: M = mean; SD = standard deviation.

**Table 2:** Descriptive statistics for ES, PWB, and LS.

	M	SD	SE	CI 95%	
				Lower Bound	Upper Bound
ES	46.28	8.78	0.52	45.251	47.322
PWB	6.08	2.06	0.12	5.846	6.331
LS	27.02	5.49	0.32	26.382	27.674

Legend: ES: emotional stability; PWB: psychological well-being; LS: life satisfaction; M = mean; SD = standard deviation; SE = standard error; CI = confidence interval.

**Table 3:** The Spearman correlations among ES, PWB, and LS.

	ES			PWB			LS		
	r	95% CI	*p* *	r	95% CI	*p* *	r	95% CI	*p* *
ES				−0.526	−0.606 to −0.436	<0.0001	0.319	0.210 to 0.421	<0.0001
PWB	−0.526	−0.606 to −0.436	<0.0001				−0.046	−0.554 to −0.365	<0.001
LS	0.319	0.210 to 0.421	<0.0001	−0.046	−0.554 to −0.365	<0.001			

Legend: ES = emotional stability; PWB= psychological well-being; LS = life satisfaction; * Spearman test *p* < 0.05 (two-tailed).

## Data Availability

Not applicable.

## Abbreviations

COVID-19	Coronavirus Disease 2019
ES	Emotional Stability
PWB	Psychological well-being
LS	Life Satisfaction
FFM	Five Factor Model, Big Five Model
DECAS	DECAS Personality Inventory
ASSET	A Shortened Stress Evaluation Tool
SWLS	Satisfaction with Life Scale
CBT	Cognitive Behavioral Therapy
ACT	Acceptance and Commitment Therapy/Training
